# 2,4,6-Triphenyl-1-hexene, an Anti-Melanogenic Compound from Marine-Derived *Bacillus* sp. APmarine135

**DOI:** 10.3390/md22020072

**Published:** 2024-01-30

**Authors:** Hye Yeon Kim, Hye-Yeon Do, Saitbyul Park, Keon Woo Kim, Daejin Min, Eun-Young Lee, Dabin Shim, Sung Yeon Cho, Jin Oh Park, Chang Seok Lee, Sang-Jip Nam, Jaeyoung Ko

**Affiliations:** 1 Department of Beauty and Cosmetic Science, Eulji University, Seongnam 13135, Republic of Korea; khh6261@naver.com (H.Y.K.); ejsdb0126@naver.com (D.S.); 2 Department of Chemistry and Nanoscience, Ewha Womans University, Seoul 03760, Republic of Korea; tkfkdgo5397@gmail.com (H.-Y.D.); younglee0124@naver.com (E.-Y.L.); 3 Basic Research & Innovation Division, AMOREPACIFIC R&I Center, Yongin 17074, Republic of Korea; sbpark0819@amorepacific.com (S.P.); djmin@amorepacific.com (D.M.); csy123@amorepacific.com (S.Y.C.); 4 Department of Natural Product Laboratory, Daebong LS Co., Ltd., Incheon 21697, Republic of Korea; kw.kim@daebongls.co.kr (K.W.K.); pjoh0303@daebongls.co.kr (J.O.P.)

**Keywords:** *Bacillus* sp., 2,4,6-triphenyl-1-hexene, marine natural products, anti-melanogenic effect

## Abstract

Although melanin protects against ultraviolet radiation, its overproduction causes freckles and senile lentigines. Recently, various biological effects of metabolites derived from marine microorganisms have been highlighted due to their potential for biological and pharmacological applications. In this study, we discovered the anti-melanogenic effect of *Bacillus* sp. APmarine135 and verified the skin-whitening effect. Fractions of APmarine135 showed the melanin synthesis inhibition effect in B16 melanoma cells, and 2,4,6-triphenyl-1-hexene was identified as an active compound. The melanogenic capacity of 2,4,6-triphenyl-1-hexene (**1**) was investigated by assessing the intracellular melanin content in B16 cells. Treatment with 5 ppm of 2,4,6-triphenyl-1-hexene (**1**) for 72 h suppressed the α-melanocyte-stimulating hormone (α-MSH)-induced intracellular melanin increase to the same level as in the untreated control group. Additionally, 2,4,6-triphenyl-1-hexene (**1**) treatment suppressed the activity of tyrosinase, the rate-limiting enzyme for melanogenesis. Moreover, 2,4,6-triphenyl-1-hexene (**1**) treatment downregulated tyrosinase, Tyrp-1, and Tyrp-2 expression by inhibiting the microphthalmia-associated transcription factor (MITF). Furthermore, 2,4,6-triphenyl-1-hexene (**1**) treatment decreased the melanin content in the three-dimensional (3D) human-pigmented epidermis model MelanoDerm and exerted skin-whitening effects. Mechanistically, 2,4,6-triphenyl-1-hexene (**1**) exerted anti-melanogenic effects by suppressing tyrosinase, Tyrp-1, and Tyrp-2 expression and activities via inhibition of the MITF. Collectively, these findings suggest that 2,4,6-triphenyl-1-hexene (**1**) is a promising anti-melanogenic agent in the cosmetic industry.

## 1. Introduction

Melanogenesis is the process of melanin production to protect the skin against the deleterious effects of ultraviolet (UV) radiation [1]. However, melanin overproduction causes freckles, senile lentigines, and other forms of melanin hyperpigmentation, which are serious aesthetic problems [2]. Melanogenesis is regulated by three key enzymes: tyrosinase and tyrosinase-related protein (TRP)-1 and -2 [3]. Tyrosinase is a rate-limiting enzyme that catalyzes two different steps in melanogenesis: the hydroxylation of L-tyrosine to 3,4-dihydroxyphenylalanine (DOPA) and the subsequent oxidation of DOPA to dopaquinine. TRP-1 catalyzes the oxidation of indol-5,6-quinone 2-carboxylic acid (DHICA) to carboxylated indole-quinone, whereas TRP-2 catalyzes the conversion of DOPA chrome to DHICA [4]. The microphthalmia-associated transcription factor (MITF) is a master transcription factor in melanogenesis that upregulates tyrosinase, TRP-1, and TRP-2 by binding to their promoter sites [5]. Thus, the upregulation or activation of these melanogenic proteins can increase melanin synthesis, whereas inhibiting these proteins could be an effective strategy for skin whitening. Several studies have been conducted to identify and isolate tyrosinase inhibitors from various natural and synthetic sources. Typically, these inhibitors are tested using monophenolic substrates such as tyrosine or diphenolic substrates such as L-dopa, with their effectiveness assessed based on dopachrome formation.

Although several synthetic anti-melanogenic agents have been reported, their applications as cosmetic ingredients are limited due to severe side effects in humans, such as high toxicity, low stability, poor skin penetration, and odor [6]. Therefore, recent studies on anti-melanogenic agents have focused on natural products that are free of side effects.

Marine microorganisms found in unexplored habitats, such as deep-sea sediments, hydrothermal vents, and Arctic and Antarctic waters, have the potential to produce novel metabolites with diverse biological activities [7]. Particularly, research evidence indicates that bacteria, fungi, and cyanobacteria, closely associated with sponges, are real producers of the bioactive compounds isolated from the sponges. For example, 140 novel bioactive compounds have been isolated from microorganisms associated with sponges between 2017 and 2022, indicating that they are a reservoir for natural products [8]. In cosmetics, marine microorganisms have attracted attention due to the presence of novel compounds with photoprotective, anti-aging, antioxidant, and skin-whitening activities [9]. Recent studies have revealed that small molecules from marine bacteria, such as pseudoalteromone A, deoxyvasicinone, (-)-4-hydroxysattabacin, and homothallin-II, exhibit anti-melanogenic effects [10,11,12,13]. Additionally, the culture extract of *Bacillus* sp. APmarine135 strongly inhibits melanogenesis in MSH-stimulated B16 melanoma cells.

**Figure 1:**
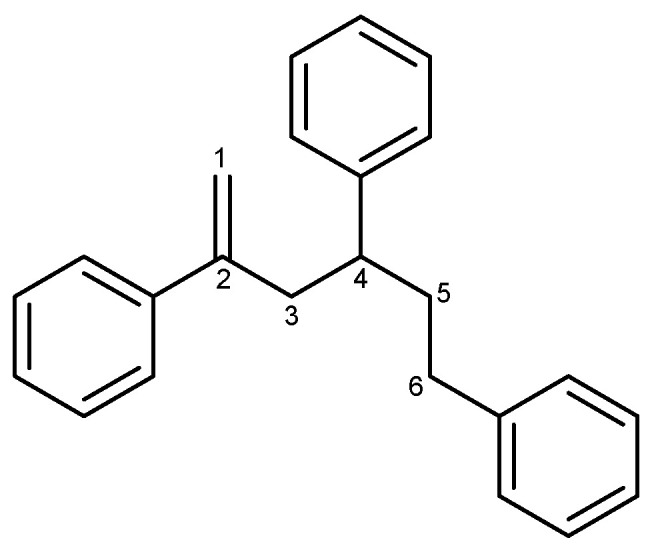
The structure of 2,4,6-triphenyl-1-hexene (**1**).

In an investigation of natural products in marine microorganisms, we collected a stony coral Scleractina sample from the Federated States of Micronesia and isolated a marine-derived *Bacillus* sp. strain called APmarine135. A high-performance liquid chromatography (HPLC)-UV-guided isolation of the culture broth of APmarine135 yielded 2,4,6-triphenyl-1-hexene as an anti-melanogenic agent (Figure 1). The 2,4,6-triphenyl-1-hexene (**1**) was previously isolated from *Phellinus pini*, a marine *Solwaraspora* sp., and exhibited estrogenic activity in MCF-7 cells [14,15,16]. However, their anti-melanogenic effects have not yet been reported. Therefore, this study aimed to investigate the inhibitory effects of 2,4,6-triphenyl-1-hexene (**1**) on melanogenesis both in vitro and in a 3D pigmented epidermis model (MelanoDerm).

## 2. Results

### 2.1. Anti-Melanogenic Evaluation of APmarine135 Fractions

**Figure 2:**
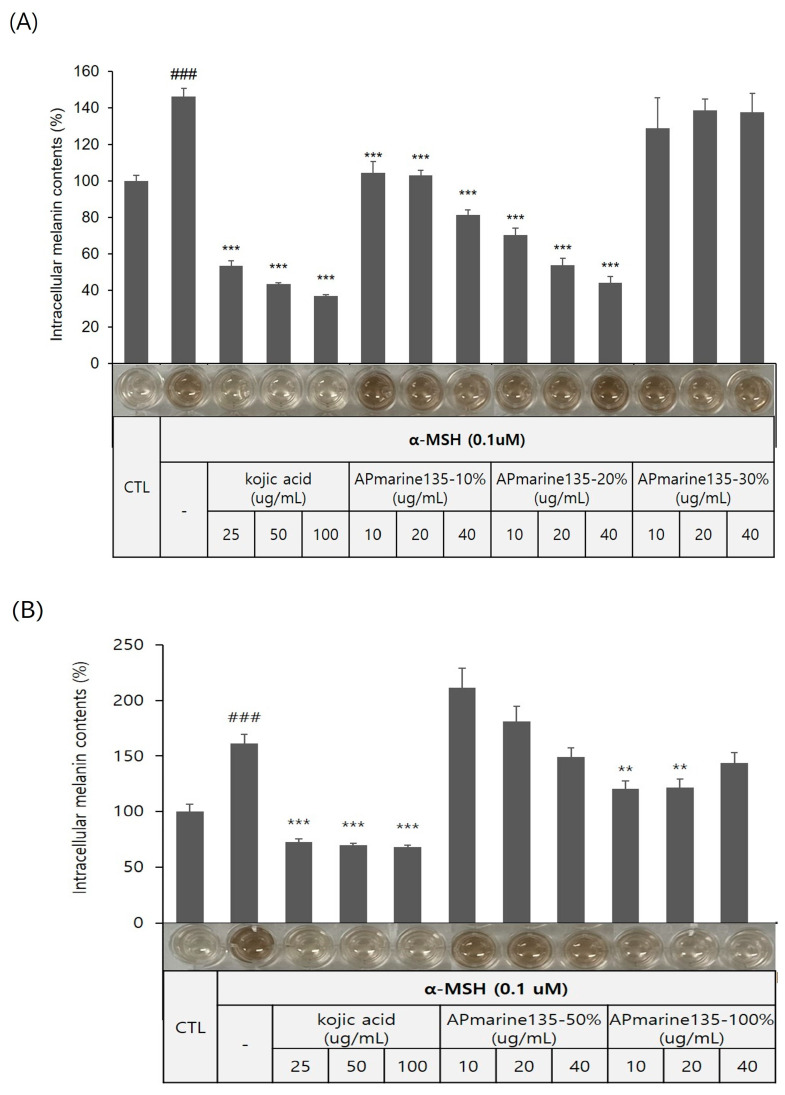
Concentration-dependent anti-melanogenic effect of APmarine135 in B16 cells. Effects of incubation with the indicated concentrations of APmarine135 for 72 h on intracellular melanin contents in α-MSH-stimulated B16 cells. APmarine135 sample conditions are 10%, 20%, 30% (**A**), 50% and 100% (**B**), rexpectively. Intracellular melanin content was determined by measuring the absorbance of B16 cell lysates at 405 nm and normalizing to the protein content, which was determined using a protein assay kit. Results are presented as mean ± standard deviation (SD), expressed as a percentage relative to the control group. (### *p* < 0.001 vs. control group, ** *p* < 0.01, *** *p* < 0.001 vs. α-MSH-treated group).

The *Bacillus* sp. APmarine135 was cultivated in 2 L of a SYP medium (10 g/L soluble starch, 4 g/L yeast, 2 g/L peptone, and 34.25 g/L sea salt in 1 L distilled water) for 3 days under constant shaking. After 60 h, sterilized a HP-20 resin (200 mL) was added to the culture medium. After mechanically stirring the culture, the HP-20 resin was collected and washed with distilled water. Thereafter, the resin was eluted with 10, 20, 30, 50, and 100% acetone. Each fraction was evaporated in a vacuum to yield APmarine135-10%, APmarine135-0%, APmarin135-30%, APmarine135-50%, and APmarie135-100%, respectively. Furthermore, we investigated the anti-melanogenic effects of each fraction and found that APmarine135-50% and APmarine135-100% inhibited melanin synthesis in B16 melanoma in a dose-dependent manner. Particularly, APmarine135-100% had the most inhibitory effect on melanogenesis (Figure 2). Therefore, we speculated that the fraction APmarine135-100% contains a potent anti-melanogenic compound.

To identify compounds responsible for the anti-melanogenic activity, we isolated compound **1** from the APmarine135_100% fraction using reverse-phase silica gel chromatography.

### 2.2. Identification of 2,4,6-Triphenyl-1-hexene

Compound **1** was isolated as a transparent oil, and its molecular formula was determined to be C_24_H_24_ based on HREIMS spectral data analysis (*m*/*z* 312.1878 [M]^+^ in HREIMS spectroscopic data). The ^1^H NMR spectrum of compound **1** displayed three sets of aromatic protons at *δ*_H_ 7.02–7.36 (15H), olefinic protons at *δ*_H_ 5.18 (1H, d, *J* = 1.6 Hz, and H-1), 4.92 (1H, dd, *J* = 3.2, 1.6 Hz, and H-1), six methylene protons at *δ*_H_ 2.86 (1H, ddd, *J* = 13.8, 7.9, 1.1 Hz, and H-3), 2.83 (1H, ddd, *J* = 13.8, 7.9, 1.1 Hz, and H-3), 2.30–2.50 (2H, m, and H-6), 2.07 (1H, dddd, *J* = 13.6, 10.4, 6.5, 4.4 Hz, and H-5), 1.92 (1H, dtd, *J* = 13.6, 10.4, 6.5 Hz, and H-5), and one methine proton at *δ*_H_ 2.71 (1H, m, H-4). The ^13^C NMR and HSQC spectroscopic data of compound **1** had 24 carbons, including 3 quaternary carbons at *δ*_C_ 146.9 (1C), 145.2 (1C), and 142.6 (1C), 15 aromatic carbons at *δ*_C_ 128.5 (4C), 128.5 (2C), 128.4 (2C), 127.9 (2C), 127.5 (1C), 126.6 (2C), 126.3 (1C), and 125.8 (1C), 2 olefinic carbons at *δ*_C_ 141.3 (1C, C-2) and 114.7 (1C, C-1), 3 methylene carbons at *δ*_C_ 43.6 (1C, C-3), 37.5 (1C, C-5), and 33.9 (1C, C-6), and 1 methine carbon at *δ*_C_ 43.8 (1C, C-4) (Figure S1). Based on the comparison with the NMR data for compound **1** in the literature [14], compound **1** was identified as 2,4,6-triphenyl-1-hexene. Although compound **1** has previously been isolated from the fungus *Phellinus pini* [14], this is the first study to isolate it from the *Bacillus* sp.

### 2.3. Inhibitory Effects of 2,4,6-Triphyenl-1-hexene on Melanin Synthesis in B16 Cells

**Figure 3:**
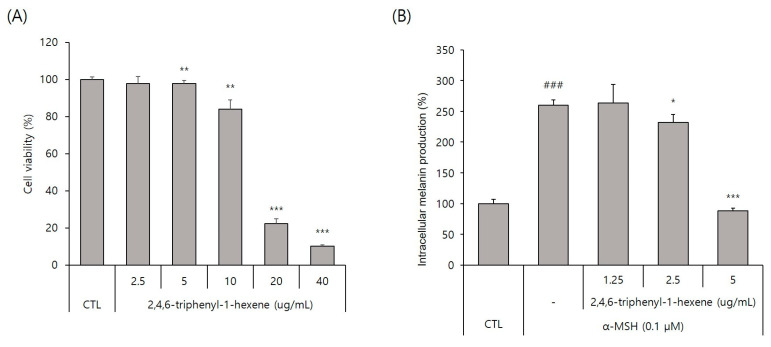

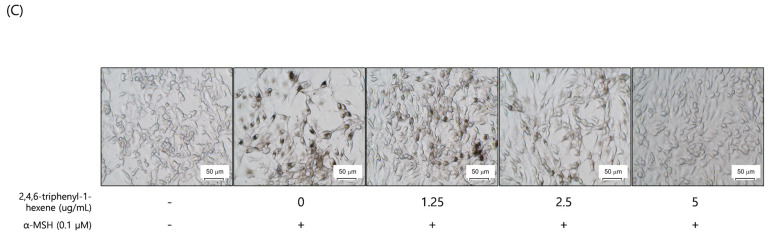
Anti-melanogenic effects of non-toxic concentrations of 2,4,6-triphenyl-1-hexene in B16 melanoma cells. (**A**) Effect of 2,4,6-triphenyl-1-hexene on B16 cell viability. B16 cells were treated with the indicated concentrations of 2,4,6-triphenyl-1-hexene for 72 h, and cell viability was estimated using a CCK-8 assay kit. (**B**) Effects of incubation with the indicated concentrations of 2,4,6-triphenyl-1-hexene for 72 h on intracellular melanin content in α-MSH-stimulated B16 cells. Intracellular melanin content was determined by measuring the absorbance of B16 cell lysates at 405 nm and normalizing the obtained values to the protein content, which was determined using a protein assay kit. Results are presented as mean ± standard deviation (SD), expressed as a percentage relative to the control group. (**C**) Microscopic images of B16 cells under the same conditions as above. Figures depict results from at least three replicate experiments. Results are presented as mean ± SD, expressed as a percentage relative to the control or α-MSH-treated group. “-” means non-treated or without any treatment, and “+” means treated with the indicated compound along with α-MSH at a concentration of 0.1 μM where indicated. Scale bar in (**C**) = 50 μm. (**A**; * *p* < 0.05, ** *p* < 0.01, *** *p* < 0.001 vs. control group, **B**; ### *p* < 0.001 vs. control group, * *p* < 0.05, *** *p* < 0.001 vs. α-MSH-treated group).

To evaluate the whitening effect of 2,4,6-triphenyl-1-hexene (**1**) on B16 cells, we first investigated the potential cytotoxicity effect of the compound. The 2,4,6-triphenyl-1-hexene (**1**) was not cytotoxic to B16 cells at concentrations of 2.5–5 ppm (Figure 3A). Additionally, 2,4,6-triphenyl-1-hexene (**1**) exerted a dose-dependent whitening effect at concentrations of 1.25–5 ppm (Figure 3B). Moreover, 2,4,6-triphenyl-1-hexene (**1**) pretreatment for 6 h significantly reduced melanin production in B16 cells stimulated with α-MSH for 72 h, which was confirmed using optical microscopy and measuring melanin content (Figure 3C).

### 2.4. Inhibitory Effect of 2,4,6-Triphyenl-1-hexene on Tyrosinase Activity and Expression in B16 Cells

**Figure 4:**
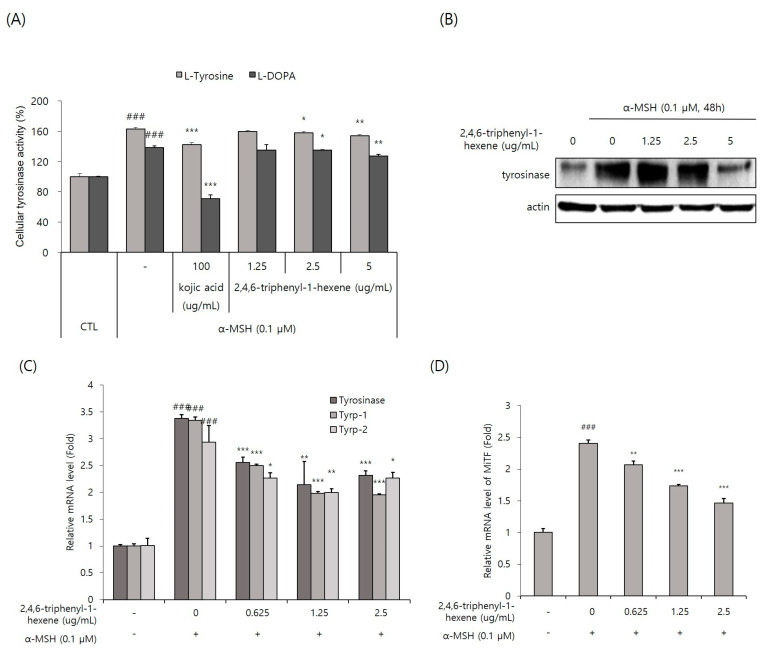
Inhibitory effect of 2,4,6-triphenyl-1-hexene on the expression of melanogenic proteins. (**A**) Cellular tyrosinase activity was spectrophotometrically measured at 475 nm, as described in Materials and Methods. (**B**) The protein expression of tyrosinase in B16 cells was determined using Western blot analysis. Cells were treated with 2,4,6-triphenyl-1-hexene for 48 h. Equal amounts of proteins were separated using 10% SDS-PAGE and detected using specific antibodies. β-Actin was detected as a loading control. (**C**,**D**) mRNA levels of melanogenic protein-encoding genes in B16 cells were determined using real-time qPCR. Cells were treated with 2,4,6-triphenyl-1-hexene for 24 h. The mRNA levels of the genes encoding tyrosinase, Tyrp-1, Tyrp-2, and the MITF were normalized to those of β-actin. The results for B16 cells are presented as means ± standard deviation (SD), expressed as a percentage relative to controls (* *p* < 0.05, ** *p* < 0.01, *** *p* < 0.001 vs. the α-MSH-treated group; ### *p* < 0.001 vs. the α-MSH-untreated control group).

Tyrosinase, the rate-limiting enzyme of the melanogenic pathway, is a copper-containing glycoprotein of approximately 60–70 kDa [4]. Thus, tyrosinase is considered a common target for therapeutic agents intended to alleviate cutaneous hyperpigmentation [2]. For this reason, many whitening materials have the function of inhibiting tyrosinase activity, lowering protein expression levels, or both. In this study, we first decided to observe whether 2,4,6-triphenyl-1-hexene affected tyrosinase activity. Cellular tyrosinase activity was evaluated using two substrates, L-tyrosine and L-DOPA, and the cell lysates of B16 cells stimulated with α-MSH. We found that intracellular tyrosinase activity was significantly, but not powerfully, inhibited via the treatment of 2,4,6-triphenyl-1-hexene (**1**) at concentrations of 2.5 and 5 ug/mL in α -MSH-stimulated B16 cells (Figure 4A). Additionally, Western blot analysis confirmed that 2,4,6-triphenyl-1-hexene (**1**) treatment decreased the expression of the tyrosinase protein in the cells (Figure 4B). Moreover, 2,4,6-triphenyl-1-hexene (**1**) pretreatment suppressed the mRNA expression of tyrosinase, Tyrp-1, and Tyrp-2 in α-MSH-stimulated B16 cells (Figure 4C). Furthermore, we examined the mRNA expression of the MITF, an important transcription factor for tyrosinase, Tyrp-1, and Tyrp-2 expression. The 2,4,6-triphenyl-1-hexene (**1**) treatment inhibited the MITF expression in B16 cells (Figure 4D). Collectively, 2,4,6-triphenyl-1-hexene (**1**) decreases tyrosinase, Tyrp-1, and Tyrp-2 expression by inhibiting the MITF inhibition, confirming the anti-melanogenesis mechanisms of 2,4,6-triphenyl-1-hexene (**1**).

### 2.5. Inhibition of Epidermal Pigmentation via 2,4,6-Triphenyl-1-hexene on a 3D Human Skin Equivalent Model

**Figure 5:**
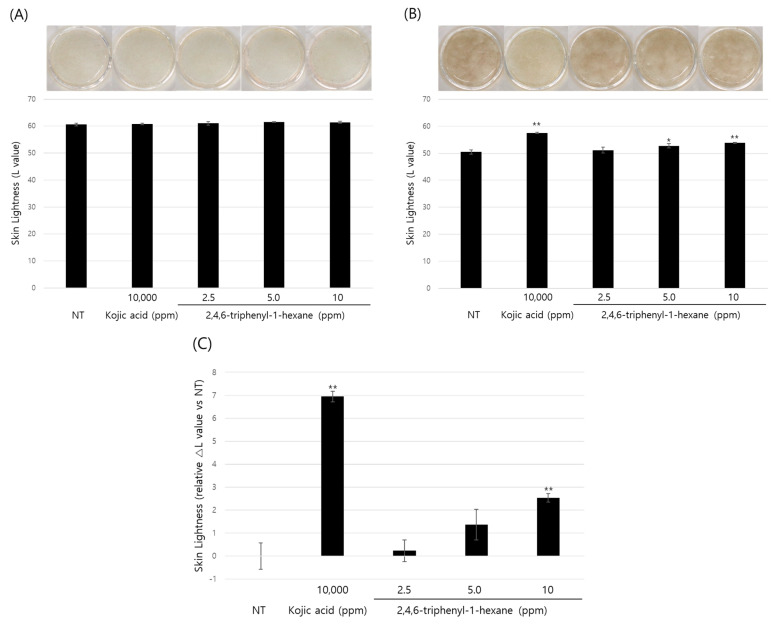

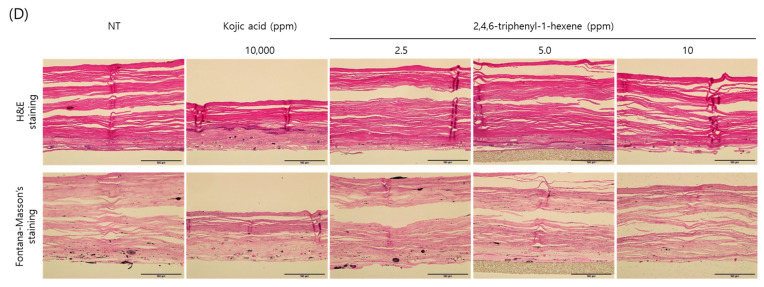
Skin-whitening effects of 2,4,6-triphenyl-1-hexene on a 3D reconstructed skin model. A human-pigmented epidermis model, MelanoDerm, was treated with different concentrations of 2,4,6-triphenyl-1-hexene for 14 days, and epidermal pigmentation was examined. Day 1 (**A**), Day 14 (**B**), changes in L value through the experimental period (**C**), and histological observations (**D**). Epidermal pigmentation was evaluated based on the L values of each tissue through image analysis, and results are presented as mean ±SD. *; *p* < 0.05 and **; *p* < 0.01 vs. the control group. Scale bar = 100 μm.

So far, we demonstrated the anti-melanogenic effect and mechanism of 2,4,6-triphenyl-1-hexene in B16 cells. However, skin pigmentation is a complex reaction consisting of melanin synthesis, transport, and transfer. Briefly, melanin produced in melanocytes at the basal layer of the epidermis is transferred to adjacent keratinocytes in the form of melanosomes. Through the sequential differentiation processes of keratinocytes, the transferred melanin is accumulated up to the outermost layer of the epidermis stratum corneum and epidermal pigmentation is completed. On the basis of this, we observed the changes in skin lightness using a human 3D pigmented epidermis model to evaluate the skin-whitening efficacy of 2,4,6-triphenyl-1-hexene. A human-pigmented epidermis model, MelanoDerm, was treated with 2,4,6-triphenyl-1-hexene (**1**) for 14 days, and skin lightness was evaluated using optical and histological examinations (Figure 5). MelanoDerm darkened with increasing culture duration due to melanin production and melanocyte transfer of melanin by melanocytes. However, 2,4,6-triphenyl-1-hexene (**1**)-treated tissues were brighter than the untreated controls. Skin brightness was also quantified through image analysis (L value). Skin brightness was continuously monitored from the start of the experiment (Day 1 (D1), Figure 5A) to the end (Day 14 (D14), Figure 5B). Furthermore, changes in skin lightness (relative △L value) showed a significant inhibitory effect on epidermal pigmentation by 2,4,6-triphenyl-1-hexene (**1**) (Figure 5C). Additionally, histological examination confirmed the whitening effect of 2,4,6-triphenyl-1-hexene (**1**) (Figure 5D). Although some degree of epidermal hyper-differentiation was observed, melanin in the epidermis decreased according to the concentration of 2,4,6-triphenyl-1-hexene (**1**). Collectively, these results demonstrate that 2,4,6-triphenyl-1-hexene (**1**) treatment may induce skin whitening by inhibiting epidermal pigmentation.

## 3. Discussion

In this study, we observed that fractions derived from the marine microorganism *Bacillus* sp. strain APmarine135 have anti-melanogenic activity in B16 cells and identified 2,4,6-triphenyl-1-hexene (**1**) as an active compound. Then, we demonstrated that 2,4,6-triphenyl-1-hexene (**1**) clearly inhibited melanin synthesis in B16 cells. Surprisingly, treatment with 2,4,6-triphenyl-1-hexene (**1**) at 5 ppm for 72 h reduced α-MSH-induced intracellular melanin production to the same level as the untreated control in B16 cells (Figure 3B).

In the current study, we used α-melanocyte-stimulating hormone (α-MSH)-stimulated B16 murine melanoma cells to find the anti-melanogenic efficacy of 2,4,6-triphenyl-1-hexene (**1**). α-MSH, known as a family of peptide hormones, stimulates the production and release of melanin via melanocytes in the skin. In B16 melanoma cells, α-MSH activates tyrosinase and melanogenesis via adenyl cyclase activation. Namely, in response to α-MSH, B16 melanoma cells underwent differentiation characterized by increased melanin biosynthesis [17]. Therefore, many studies have conducted experiments using B16 cell lines stimulated with α-MSH to verify the inhibition of melanin production via whitening materials [17,18,19,20]. In particular, referring to these reports, since α-MSH maximizes melanin production in B16 around 72 h, the inhibitory efficacy of melanin production via 2,4,6-triphenyl-1-hexene was also verified at 72 h in α-MSH-stimulated B16 cells in this study. In addition, in order to examine the inhibitory effect of 2,4,6-triphenyl-1-hexene (**1**) on the potent targets involved in melanin production, the current experiments were performed at 48 h for the protein level and 24 h for the mRNA level. Although the general in vitro experiment using the α-MSH-stimulated B16 cells is widely accepted, it was thought that it would be more realistic to verify the anti-melanogenic efficacy using human melanocytes. However, experimental data using human cells were not obtained in this study. Instead, we obtained more meaningful and realistic whitening efficacy data in this study using a 3D reconstructed skin model.

In general, many anti-melanogenic compounds based on tyrosinase inhibition are characterized by having tyrosine moiety. However, despite its anti-melanogenic activity, 2,4,6-triphenyl-1-hexene (1) does not contain any phenolic group derived from tyrosine. To the best of our knowledge, 2,4,6-triphenyl-1-hexene (**1**) is the first class of anti-melanogenic inhibitors without any tyrosine moiety in its structure. The ability of 2,4,6-triphenyl-1-hexne (**1**) to inhibit melanin production was also confirmed using color photographs of B16 cells. To define the underlying molecular mechanisms of 2,4,6-triphenyl-1-hexene (**1**) in anti-melanogenesis, we first confirmed that 2,4,6-triphenyl-1-hexene (**1**) suppressed the activity of tyrosinase, the rate-limiting enzyme for melanogenesis. As shown in Figure 4A, 2,4,6-triphenyl-1-hexene (**1**) slightly but significantly reduced the activity of tyrosinase. However, because it did not strongly inhibit tyrosinase activity, it was difficult to consider that inhibition of tyrosinase activity via 2,4,6-triphenyl-1-hexene (**1**) would have played an important role in melanin production. In addition, it was observed that the results of tyrosinase activity inhibition were slightly different depending on whether L-tyrosine and L-DOPA were used as substrates. This may be predicted as a result of the substrate concentration or solvent due to limitations in in vitro experimental conditions, but there is also the possibility that inhibition of tyrosinase activity via 2,4,6-triphenyl-1-hexene (**1**) may differ depending on the substrate on which tyrosinase acts during the melanin production process. Therefore, we conducted additional experiments focusing on the inhibition of protein expression rather than the efficacy in inhibiting tyrosinase activity via 2,4,6-triphenyl-1-hexene (**1**). As expected, 2,4,6-triphenyl-1-hexene (**1**) induced a reduction in tyrosinase, Tyrp-1, and Tyrp-2 expression through the MITF inhibition, confirming that this is the mechanism by which melanin production is suppressed (Figure 4B,C). Interestingly, 2,4,6-triphenyl-1-hexene (**1**) significantly suppressed both tyrosinase expression and activity. These results imply that 2,4,6-triphenyl-1-hexene (**1**) could act as a powerful tyrosinase inhibitor like kojic acid or arbutin, although toxicity must be considered under experimental conditions. In addition, further study will be needed to determine whether signaling pathways such as cAMP-PKA-CREB, which are upstream signaling pathways that regulate MITF-tyrosinase signaling, are inhibited.

To confirm the anti-melanogenic activity of 2,4,6-triphenyl-1-hexene (**1**), we further evaluated the skin-whitening effect of 2,4,6-triphenyl-1-hexene (**1**) using the human three-dimensional (3D) pigmented epidermis model, MelanoDerm. MelanoDerm is a type of reconstructed human epidermis model and is widely used to verify the skin-whitening efficacy of substances due to its similarity in histological and physiological characteristics for epidermal pigmentation, especially in pre-clinical studies [21,22].

In this study, the morphology and epidermal pigmentation were normal during the whole period of study (Figure 5A,B, macroscopic view). However, epidermal hyper-differentiation was also observed through histological observation (Figure 5D). This is a unique feature of MelanoDerm that we have observed in previous studies [10,23,24,25]. However, it is also necessary to comprehensively examine whether this phenomenon affected the epidermal pigmentation. Macroscopic observations clearly demonstrated the difference in pigmentation of the epidermis according to the substance treatment. Epidermal pigmentation was significantly suppressed via kojic acid as a positive control, and the concentration-dependent suppression of epidermal pigmentation was also observed via the test substance. Furthermore, the Fontana–Masson’s (F-M) staining results clearly demonstrate the difference in the amount of melanin accumulated in the epidermis. Therefore, although there was some degree of epidermal hyper-differentiation, these results show that epidermal pigmentation was normal in this model. However, because it is not sufficient to demonstrate the viability of melanocytes through F-M staining alone, we are planning to reinforce this through follow-up studies. In addition, many other cells in the skin, such as immune cells and dermal fibroblasts, could affect skin pigmentation [26,27,28,29]. However, MelanoDerm consists only of keratinocytes and melanocytes. So, it also has limitations in representing the complex pigmentation reactions that occur in native skin.

Therefore, it is noteworthy that we demonstrate the potential skin-whitening efficacy of 2,4,6-triphenyl-1-hexene (**1**) for the first time. However, we will verify the mechanism and confirm the efficacy of 2,4,6-triphenyl-1-hexene (**1**) through follow-up studies. First, for a deeper understanding, it will be necessary to determine how 2,4,6-triphenyl-1-hexene (**1**) inhibits the MITF-tyrosinase signaling pathway. Then, to confirm the skin-whitening effect, both 2,4,6-triphenyl-1-hexene (**1**) alone or formulations containing it will be applied to the upper part of MelanoDerm or ex vivo human skin. Finally, the skin-whitening effect of 2,4,6-triphenyl-1-hexene (**1**) will be verified through clinical studies.

## 4. Materials and Methods

### 4.1. General Experimental Procedure

Low-resolution LC/MS measurements were performed using the Agilent Technologies 1260 quadrupole (Agilent Technologies, Santa Clara, CA, USA) and Waters Micromass ZQ LC/MS system (Waters Corp, Milford, MA, USA) using a reversed-phase column (Phenomenex, Torrance, CA, USA) (Phenomenex Luna C18 (2) 100 Å, 50 mm × 4.6 mm, 5 µm) at a flow rate of 1.0 mL/min at the National Research Facilities and Equipment Center (NanoBioEnergy Materials Center) at Ewha Womans University. The NMR spectra were obtained using an Agilent NMR spectrometer (Agilent, Santa Clara, CA, USA) at 400 MHz for ^1^H and at 100 MHz for ^13^C using the signals of the residual solvent as internal references (δ_H_ 7.24 ppm and δ_C_ 77.2 ppm for deuterated chloroform [CDCl_3_]). High-resolution EI-MS spectra were acquired using a JEOL JMS-AX505WA mass spectrometer (JEOL Ltd., Tokyo, Japan) at Seoul National University.

### 4.2. Collection and Phylogenetic Analysis of Strain APmarine135

The marine-derived *Bacillus* sp. strain APmarine135 was isolated from a stony coral, Scleractinia, collected from the Federated States of Micronesia. The strain was identified as a *Bacillus* sp. based on 16S rRNA gene sequence analysis (accession number NR_025264.1).

### 4.3. Cultivation, Extraction, and Isolation

The *Bacillus* sp. APmarine135 was cultivated in 54 L of a SYP medium (10 g/L of soluble starch, 4 g/L of yeast, 2 g/L of peptone, and 34.25 g/L of sea salt in 1 L of distilled water) for 3 days at 27 °C under constant shaking at 80 rpm. After 60 h, a sterilized HP-20 resin (5.4 L) was added, followed by further incubation for 12 h, after which the resin was collected and washed four times (30 min each) with 54 L of 20% acetone (H_2_O/acetone, 80/20 by volume). Finally, the resin was eluted with 54 L of 99.5% acetone for 24 h under constant shaking. Acetone was removed under reduced pressure, and the resulting crude extract (3.5 g) was fractionated via medium-pressure liquid chromatography (MPLC) using a silica column. The mobile phases A and B were n-hexane and chloroform, respectively. The samples were run for 60 min at a flow rate of 20 mL/min with the following gradient program for solvent B: 0 to 10.9 min, 0%; 10 to 21.8 min, 0 to 11.9%; 21.8 to 26 min, 11.9%; 26 to 37 min, 11.9 to 26%; 37 to 43 min, 26%; 43 to 52.6 min, 26 to 100%; and 52.6 to 60 min and 100% to obtain fractions M1–M7. The sample was eluted with 100% methanol for 25 min and labeled M8. Fraction M3 was purified using preparative HPLC (Phenomenex Luna C18 [2] 100 Å, 250 mm × 21.2 mm). The mobile phases A and B were water and acetonitrile, respectively. The sample was run for 30 min at a flow rate of 15 mL/min using the following gradient program for solvent B: 0 to 5 min, 75%; and 5 to 25 min, 75 to 95%; 25 to 30 min, 95% to yield 2,4,6-triphenyl-1-hexene (**1**, *t_R_* 20.0 min, 36 mg).

The 2,4,6-triphenyl-1-hexene (**1**): transparent oil; ^1^H (400 MHz, CDCl_3_): δ_H_ 7.02–7.36 (15H), 5.18 (1H, d, *J* = 1.6 Hz, H-1), 4.92 (1H, dd, *J* = 3.2, 1.6 Hz, H-1), 2.86 (1H, ddd, *J* = 13.8, 7.9, 1.1 Hz, H-3), 2.83 (1H, ddd, *J* = 13.8, 7.9, 1.1 Hz, H-3), 2.30–2.50 (2H, m, H-6), 2.71 (1H, m, H-4), and 2.07 (1H, dddd, *J* = 13.6, 10.4, 6.5, 4.4 Hz, H-5), 1.92 (1H, dtd, *J* = 13.6, 10.4, 6.5 Hz, and H-5); ^13^C (100 MHz, CDCl_3_) δ_C_ 146.9 (1C), 145.2 (1C), 142.6 (1C), 141.3 (1C, C-2), 128.5 (4C), 128.5 (2C), 128.4 (2C), 127.9 (2C), 127.5 (1C), 126.6 (2C), 126.3 (1C), 125.8 (1C), 114.7 (1C, C-1), 43.8 (1C, C-4), 43.6 (1C, C-3), 37.5 (1C, C-5), and 33.9 (1C, C-6); HR-EI-MS m/z 312.1878 [M]+ (calculated for C_24_H_24_, 312.1878).

### 4.4. Cell Culture

The B16 mouse melanoma cell line was cultured in Dulbecco’s modified Eagle’s medium (DMEM; Welgene, Gyeongsan-si, Republic of Korea) supplemented with 5% of a fetal bovine serum (FBS; ATCC, Manassas, VA, USA) and a 1% penicillin–streptomycin mixture (Lonza, Basel, Switzerland). B16 cells were cultured at 37 °C in a humidified environment under 5% CO_2_ and 95% air.

### 4.5. Cell Viability Assay

Cell viability was determined using the Quanti-MAX™ WST-8 Cell Viability Assay Kit (Biomax, Seoul, Republic of Korea) according to the manufacturer’s protocols. Cells were seeded in a 96-well plate (8000 cells/well) for 24 h, and the culture medium was replaced with a fresh culture medium containing 2,4,6-triphenyl-1-hexene diluted to the indicated concentrations. After 72 h, cell viability was assessed by replacing the medium with an appropriate medium containing a 10% WST-8 solution. The plate was incubated for 1 h, and absorbance was measured at 450 nm using a Microplate Reader (BioTek, Winooski, VT, USA). Cell viability was calculated using the following formula: cell viability (%) = (optical density (450 nm) of the experimental group/optical density of the control group) × 100.

### 4.6. Measurement of Melanin Content

To quantify melanin content, B16 cells (2 × 10^4^ cells/well in 48-well plates) were cultured for 24 h, followed by pretreatment with increasing concentrations of 2,4,6-triphenyl-1-hexene (**1**) for 6 h. Thereafter, α-MSH (0.1 μM) was added to induce melanin production in a phenol red-free cell culture medium. After 72 h, 1N NaOH was added to each well to measure the intracellular melanin content, and the plate was heated to 60 °C for 30 min. The resulting lysate was aliquoted into a 96-well plate, and the absorbance was determined at 405 nm. Intracellular melanin content was normalized to the total protein content. Melanin levels were calculated via comparison with those of the corresponding controls and are shown as percentages.

### 4.7. Cellular Tyrosinase Activity Assay

Cellular tyrosinase activity was estimated by measuring oxidase activity. Briefly, B16 cells were lysed in 1 mL of a sodium phosphate buffer (0.1 M, pH 6.8) containing 1% Triton X-100 for 20 min, followed by centrifugation at 13,000 rpm for 20 min at 4 °C. The supernatant and substrate solution (10 mM L-DOPA or 0.03% L-tyrosine) were dispensed in equal volumes into a 96-well plate, followed by the addition of 2,4,6-triphenyl-1-hexene (**1**). The cellular tyrosinase activity was determined at 475 nm using a spectrophotometer at least 5 times over 10 at an incubation temperature of 37 °C.

### 4.8. Western Blot Analysis

Briefly, B16 cells were cultured in 6-well plates, followed by treatment with 2,4,6-triphenyl-1-hexene (**1**). The next day, the cells were washed twice with cold PBS, and total intracellular proteins were extracted on ice for 20 min using the RIPA buffer 1X (Cell Signaling Technology, Danvers, MA, USA) supplemented with a 1:200 dilution of a protease inhibitor cocktail III and phosphatase inhibitor cocktail set III (Calbiochem Biosciences, La Jolla, CA, USA). After centrifugation at 13,000 rpm and 4 °C, the supernatant was collected, and the total protein content was determined using a Pierce bicinchoninic acid (BCA) protein assay kit. Equal amounts of soluble proteins were resolved using 10% SDS-PAGE and transferred to a nitrocellulose membrane (BioRad, Hercules, CA, USA) in a cold transfer buffer (25 mM Tris, 192 mM glycine, and 20% (*w*/*v*) MeOH) for 90 min at 280 mA. Thereafter, the membranes were blocked for at least 1 h with TBS 1X supplemented with a 5% blotting-grade blocker (BioRad) and incubated overnight at 4 °C with primary antibodies diluted to the appropriate concentration (as per the provided data sheet) in 1X TBS. Primary antibodies against β-actin (#ab3280) and tyrosinase (#ab52493) were purchased from Abcam (Cambridge, UK). Following overnight incubation, the membranes were further incubated with secondary antibodies and enhanced using a Clarity™ Western ECL substrate (ECL solution; BioRad). Goat anti-rabbit IgG (HRP) (#ab6721) and rabbit anti-mouse IgG (HRP) (#ab6728) secondary antibodies were purchased from Abcam (Cambridge, UK). Images of the blotted membranes were captured using an iBright™ CL750 Imaging System (Invitrogen, Carlsbad, CA, USA). The expression levels of the proteins were normalized to those of endogenous actin.

### 4.9. Real-Time PCR

To determine the mRNA expression levels of melanogenesis-related genes, B16 cells were cultured in 6-well plates and treated with 2,4,6-triphenyl-1-hexene (**1**). The next day, the cells were washed twice with cold PBS, and the total RNA was extracted from cell lysates using a TRIzol reagent (Thermo Fisher Scientific, Waltham, MA, USA) according to the manufacturer’s instructions. Complimentary DNA was synthesized from total mRNA using the GoScript™ Reverse Transcription System (Promega, Madison, WI, USA) and a thermal cycler (T-100; BioRad). The PCR amplification of the target cDNA was performed on the CFX Connect Optics Module (BioRad) using iQ SYBR^®^ Green Supermix (BioRad) and specific primers. The mRNA levels of the target genes were normalized to those of β-actin. Specific primers were purchased from BioRad Laboratories.

### 4.10. Evaluation of Skin-Whitening Efficacy Using a 3D Reconstructed Skin Model

The MelanoDerm (MEL-300-B, MatTek Corp., Ashland, MA, USA) was incubated in an EPI-100-NMM-113-PRF medium (MatTek Corp., Ashland, MA, USA) at 37 °C in a humidified 5% CO_2_ incubator. The MelanoDerm used in this experiment was MEL-300-B, and it was manufactured using keratinocytes and melanocytes derived from the neonatal foreskin of African-American babies. MelanoDerm was treated with different concentrations of 2,4,6-triphenyl-1-hexene (**1**) every other day for 14 days. Epidermal pigmentation was examined via optical and histological observations. Epidermal pigmentation was calculated by comparing variations in L values (a lightness/darkness index) on days 1 and 14 and estimating the difference between them (∆L). For histological examination, tissues were fixed in 10% neutral buffered formalin (BBC Biochemical, Mount Vernon, WA, USA), embedded in paraffin, and cut into 5 µm sections. Thereafter, the sections were stained with hematoxylin and eosin (H&E) and Fontana–Masson’s (F-M) and imaged.

## 5. Conclusions

In this study, we identified 2,4,6-triphenyl-1-hexene (**1**) as a potent skin-whitening agent derived from the marine microorganism of APmarine 135. We demonstrated that 2,4,6-triphenyl-1-hexene (**1**) suppressed melanin synthesis and simultaneously reduced both the activity and expression of tyrosinase in B16 cells. Then, we verified its skin-whitening effect using a 3D human-pigmented epidermis model, MelanoDerm. Based on these results, we conclude that marine microorganisms are a good source of biologically active compounds and further suggest that 2,4,6-triphenyl-1-hexene (**1**) is a promising anti-melanogenic agent in the cosmetic and pharmaceutical field.

## Data Availability

Data are contained within the article and supplementary materials.

## Supplementary Materials

The following supporting information can be downloaded at: https://www.mdpi.com/article/10.3390/md22020072/s1, Figure S1: ^1^H NMR spectrum of 2,4,6-triphenyl-1-hexene (1); Figure S2: ^13^C NMR spectrum of 2,4,6-triphenyl-1-hexene (1).
