# Supplementary material for: 2,4,6-Triphenyl-1-hexene, an Anti-Melanogenic Compound from Marine-Derived Bacillus sp. APmarine135
Source: Mar Drugs. 2024 Jan 30;22(2):72. doi: 10.3390/md22020072 (PMC10890162; doi:10.3390/md22020072)

Supporting Information

2,4,6-Triphenyl-1-hexene, an Anti-Melanogenic Compound from Marine-Derived *Bacillus* sp. APmarine135

Hye Yeon Kim [1,†], Hye-Yeon Do [2,†], Saitbyul Park [3], Keon Woo Kim [4], Daejin Min [3], Eun-Young Lee [2], Dabin Shim [1], Sung Yeon Cho [3], Jin Oh Park [4], Chang Seok Lee [1,*], Sang-Jip Nam [2,*] and Jaeyoung Ko [3,*]

[1] Department of Beauty and Cosmetic Science, Eulji University, Seongnam 13135, Republic of Korea; khh6261@naver.com (H.Y.K.); ejsdb0126@naver.com (D.S.)
[2] Department of Chemistry and Nanoscience, Ewha Womans University, Seoul 03760, Republic of Korea; tkfkdgo5397@gmail.com (H.-Y.D.); younglee0124@naver.com (E.-Y.L.)
[3] Basic Research & Innovation Division, AMOREPACIFIC R&I Center, Yongin 17074, Republic of Korea; sbpark0819@amorepacific.com (S.P.); djmin@amorepacific.com (D.M.); csy123@amorepacific.com (S.Y.C.)
[4] Department of Natural Product Laboratory, Daebong LS Co., Ltd., Incheon 21697, Republic of Korea; kw.kim@daebongls.co.kr (K.W.K.); pjoh0303@daebongls.co.kr (J.O.P.)
* Correspondence: cslee2010@eulji.ac.kr (C.S.L.); sjnam@ewha.ac.kr (S.-J.N.); jaeyoungko@amorepacific.com (J.K.)
† These authors contributed equally to this work.

Table of Contents

Figure S1 ^{1}H NMR spectrum (400 MHz, $CDCl_3$) of 2,4,6-triphenyl-1-hexene (**1**) **S3**

Figure S2 ^{13}C NMR spectrum (100 MHz, $CDCl_3$) of 2,4,6-triphenyl-1-hexene (**1**) **S4**

Figure S1 ^{1}H NMR spectrum (400 MHz, $CDCl_3$) of 2,4,6-triphenyl-1-hexene (**1**)

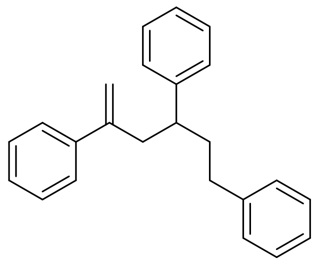

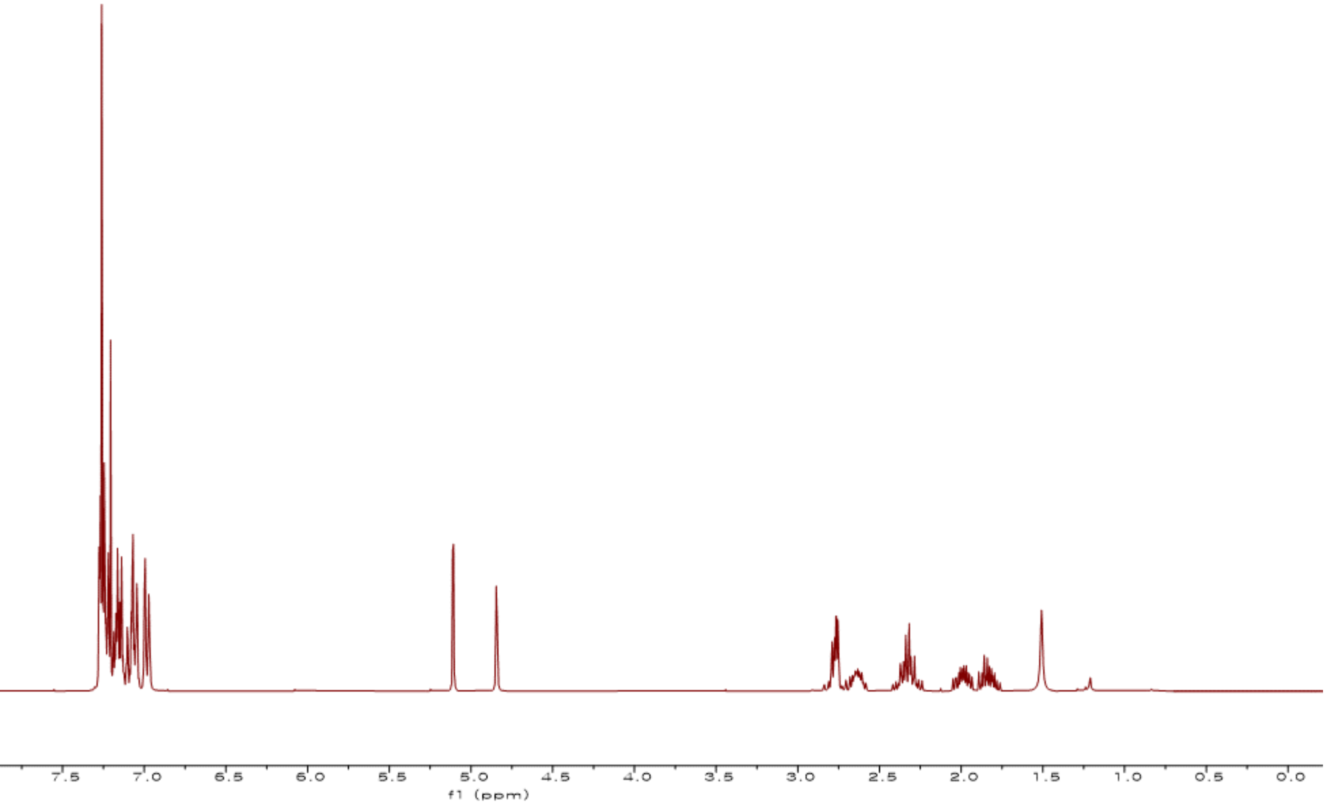

Figure S2 ^{13}C NMR spectrum (100 MHz, $CDCl_3$) of 2,4,6-triphenyl-1-hexene (**1**)

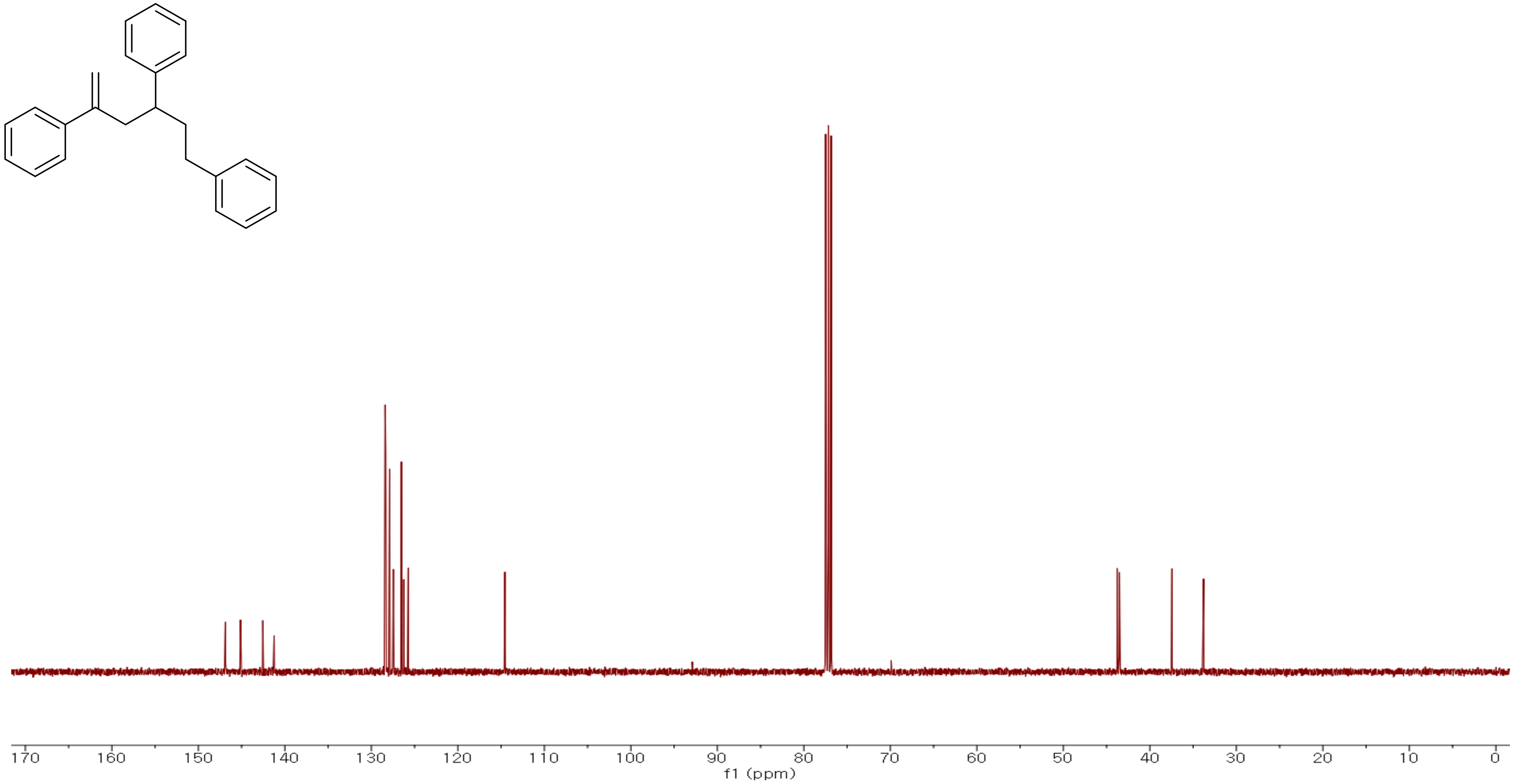

Supplement: Supplementary file 1 [file marinedrugs-22-00072-s001.zip › marinedrugs-2731685-supplementary.pdf]
